# Evaluation of serum total antioxidant level, nutritional status and Mediterranean diet adherence of adult women with rheumatoid arthritis: a case–control study

**DOI:** 10.1017/S0007114524003386

**Published:** 2025-01-28

**Authors:** Cansu Bekar, Berkan Armagan, Alper Sari, Aylin Ayaz

**Affiliations:** 1 Department of Nutrition and Dietetics, Hacettepe University, Ankara, Turkey; 2 Department of Nutrition and Dietetics, Burdur Mehmet Akif Ersoy University, Burdur, Turkey; 3 Department of Rheumatology, Hacettepe University, Ankara, Turkey

**Keywords:** Rheumatoid arthritis, Serum antioxidants, Dietary antioxidants, Nutritional status, Mediterranean diet

## Abstract

Rheumatoid arthritis (RA) is characterised by chronic inflammation in joints. Obesity, stress, being women and dietary pattern are important in pathogenesis. The joint damage in RA is accelerated by oxidative stress. The aim of this study was to examine the serum total antioxidant level, nutritional status and Mediterranean diet adherence of adult women with RA. Thirty-five adult women RA patients and thirty-five healthy control participated in this study (45·4 ± 11·61 and 42·5 ± 8·50 years, respectively). Nutritional status, physical activity levels and adherence to the Mediterranean diet were questioned. Physicians assessed the disease activity score of patients with RA. Serum total antioxidant and oxidant status were analysed. The serum total antioxidant status of the control group was higher, whereas the oxidative stress index and total oxidant status were lower than that of the RA group. Dietary protein, fibre, EPA, retinol, Fe, Zn and total antioxidant intake in the RA group were lower than in the control group (*P* < 0·05). Individuals with higher fibre intake showed a significantly lower risk for RA after adjusting for potential confounding factors (OR = 0·845, 95 % CI = 0·773–0·923, *P* < 0·001). The mean physical activity level of the control group was higher than that of the RA group (1·59 ± 0·10 and 1·53 ± 0·13, respectively) (*P* = 0·01). In conclusion, serum antioxidant parameters and dietary antioxidant intake are decreased in patients with RA. Therefore, medical treatment for these patients should be supplemented with medical nutrition therapy to achieve optimal nutritional status.

Rheumatoid arthritis (RA), a chronic inflammatory disease that can cause permanent disability, is characterised by progressive joint damage. Among rheumatic and musculoskeletal diseases, RA is the most prevalent systemic autoimmune disease^(1)^. While the exact cause of RA pathogenesis is still unknown, a combination of genetic, epigenetic and environmental factors are involved. Smoking, obesity, stress, being women and dietary factors such as Western-style dietary patterns affecting the microbiota are important in pathogenesis^(2,3)^.

It is noted that inflammation characterised by RA may cause an increase in oxidative and lipid peroxidation while decreasing levels of antioxidant and antioxidant defences^(4)^. When T cells and macrophages are activated at the site of inflammation, there is a noticeable rise in oxygen consumption. Overuse of oxygen causes reactive oxygen species, which rise to oxidative stress^(5)^. Reactive oxygen species can react with DNA, lipids and proteins, causing the destruction of hyaluronic acid and deterioration of membrane function by oxidation of collagen, proteoglycans, protease inhibitors and membrane fatty acids. Thus, it is believed that oxidative stress and lipid peroxidation are crucial to the pathophysiology of RA. Metabolic activity, pollution, diet and microbiota imbalances can lead to overproduction of ROS^(6,7)^. In a recent meta-analysis, it was reported that natural antioxidants can reduce systemic and local oxidative stress, increase serum antioxidant levels and reduce damage in RA^(8)^.

Antioxidants reduce chain initiation and/or stop the chain propagation response by scavenging or directly inhibiting the toxic non-radicals or active free radicals. As a result, they significantly mitigate the effects of oxidative stress in a variety of diseases, including cancer, atherosclerosis, neurodegenerative diseases, diabetes and RA^(9)^. Enzymatic and non-enzymatic antioxidants make up the two main categories of the human antioxidant system. Enzymatic antioxidants include glutathione reductase, catalase, glutathione peroxidase and superoxide dismutase. Non-enzymatic endogenous antioxidants include vitamins, peptides (like glutathione), nitrogen compounds (like uric acid) and enzyme cofactors^(10)^. The endogenous antioxidant system, although remarkably effective, is insufficient to sustain low levels of free radicals in humans. Consequently, vitamins A, C and E, as well as Se and Zn, which have antioxidant properties in RA patients, should be taken at the recommended level^(11)^.

The possible adverse effects and limited efficacy of drugs have led to a growing interest in new therapeutic approaches, such as diet modification^(4)^. While high fibre, ω-3 PUFA, tocopherols, carotenoids, phenolic compounds, vitamin C and vitamin D are protective for RA with their anti-inflammatory and antioxidant properties, high consumption of red meat, *trans*-fatty acids, nitrites, salt, refined sugar and low intake of ω-3 fatty acids have negative effects on RA^(12)^. It has been reported that dietary patterns and nutritional supplements may complement standard RA treatment due to their potential protective effects. Therefore, promoting a healthy lifestyle and nutrition is of great importance for patients with RA^(13)^.

Mediterranean diet is inversely associated with the risk of RA^(14)^. Some studies have shown that the Mediterranean diet may help improve disease activity scores in patients with RA. In addition, it has been reported that consumption of vegetables, fruits and legumes, which are components of the Mediterranean diet, is negatively associated with the disease score, while red meat, butter and sweetened beverages and pastries are positively associated^(15,16)^. Mediterranean diet may also slow the progression of RA by reducing oxidative stress processes^(17)^. While it has been reported that adherence to the Mediterranean diet is negatively associated with coagulation and inflammatory markers, serum lipid level and blood pressure, it is positively associated with serum total antioxidant capacity^(18,19)^. Plant-based foods like fruits, vegetables, grains, legumes, oilseeds and olives are abundant in the Mediterranean diet. The characteristics of this diet are low consumption of red meat, moderate consumption of dairy products, poultry, eggs and high consumption of fish and seafood^(20)^. The Mediterranean diet is rich in antioxidants such as ω-3 PUFA, oleic acid, vitamin E, carotenoids and flavonoids^(17)^.

In previous studies, antioxidant intake or serum antioxidant status of RA patients has generally been evaluated with a single parameter, and the study evaluating the relationship between serum total antioxidant, oxidant status and dietary components and diet quality together is limited. Since the study results show contradictions because of the high heterogeneity of the disease, new studies are needed to reach more robust conclusions about specific dietary interventions aimed at improving RA outcomes. Because it is not practical to measure antioxidant molecules separately and antioxidants can have synergistic effects, it is important to evaluate the total antioxidant and oxidant status. For these reasons, this study aimed to evaluate both the dietary and serum antioxidant status of patients with RA at the total level and also to evaluate their compliance with the Mediterranean diet instead of considering the diet component alone.

## Materials and methods

### Participants

Participants in this study included thirty-five adult women with RA and thirty-five adult healthy women controls. This study was conducted at the Department of Rheumatology, Hacettepe University, Ankara, Turkey. Individuals in the case group were selected from patients diagnosed with RA according to the diagnostic criteria of the American College of Rheumatology/European League Against Rheumatism^(21)^. The control group was included in the study with a similar distribution to the case group in terms of age and BMI. Individuals who did not have chronic diseases and did not take medication regularly were included in the control group. The sample size calculation of the study was determined as thirty-five patients and thirty-five controls with a significance level of 0·05 and a power of 0·90 in the G-Power programme. All the patients and controls did not take any antioxidant supplements, and they were also not smokers or alcohol consumers for the last year. This study was conducted according to the guidelines laid down in the Declaration of Helsinki, and all procedures involving human subjects/patients were approved by the Hacettepe University Clinical Research Ethical Board with decision number GO 17/213–26. Written informed consent was obtained from all subjects/patients.

### Data collection

The individuals’ sociodemographic characteristics, 24-h food consumption records for 2 d and physical activity status were recorded face-to-face with a survey form. The daily nutrient and energy intake were determined by the Nutrition Information System (BeBIS) 8·1 computer package programme. Dietary total antioxidant capacity was calculated by the ferric reducing antioxidant power database of foods, which was published by Carlsen *et al.* ^(22)^.

Adherence to the Mediterranean diet was determined by the Mediterranean diet adherence score, developed by Schröder *et al.* ^(23)^ and validated in Turkish by Bekar and Goktas^(24)^. The Mediterranean diet adherence score examines the consumption habits of foods in two questions and the frequency of food consumption in twelve questions. The total score ranges from 0 to 14. A score 0 shows low adherence, and a score 14 shows the highest adherence to the Mediterranean diet. The Mediterranean diet adherence score is classified as low for less than 5, moderate for 6–9 and high for ≥ 9 points^(23)^.

The physical activity levels of individuals were questioned using a retrospective 24-h recall method. The energy spent for each activity was calculated by multiplying the activity-specific physical activity rate, the duration of the activity (minutes) and the BMR per hour. Total energy expenditure was found by adding up the energy spent for each activity. The calculation of an individual’s physical activity levels involved dividing their total energy expenditure by their BMR. Physical activity level value was classified as sedentary or light activity lifestyle (1·40–1·69), active or moderately active lifestyle (1·70–1·99) and vigorous or vigorously active lifestyle (2·00–2·40)^(25)^. The height (m) and body weight (kg) of all individuals were measured, and their BMI (kg/m^2^) was calculated. Participants are divided into four classes: underweight (<18·5 kg/m^2^), normal weight (18·5–24·9 kg/m^2^), overweight (25·0–29·9 kg/m^2^) and obese (≥30·0 kg/m^2^)^(26)^. The disease severity of the individuals in the RA group was evaluated with the disease activity score 28 (DAS28). This score evaluates twenty-eight joints and is calculated by the number of swollen joints and tender joints, erythrocyte sedimentation rate or C-reactive protein values and the visual scale results that the patient uses to evaluate her condition in general. The Visual Analog Scale was used for the patient’s general evaluation of herself. The level of disease activity can be classified as low (DAS28 ≤ 3·2), moderate (3·2 < DAS28 ≤ 5·1) or high (DAS28 > 5·1)^(27)^.

### Analysis of total antioxidant and total oxidant status

Samples of fasting blood were collected in order to analyse the serum total antioxidant status (TAS) and total oxidant status (TOS). The blood samples were centrifuged for 10 min at 4 °C and 2000 ***g*** and stored at –80 °C until analysis day. All antioxidant molecules in the samples are based on the reduction of the coloured ethylbenzothiazoline sulfonic acid cationic radical, and the colour radical is decolourised in proportion to the total amount of antioxidant molecules. The total antioxidant level is proportional to the change in absorbance measured at a wavelength of 660 nm. This process is performed with an automatic analyser and calibrated with Trolox. Results are expressed in mmol Trolox Equivalent/L^(28)^. The ferrous ion–o-dianisidine complex is oxidised to ferric ions by the oxidants in the samples, which is the basis for TOS measurement. Ferric ions combine with chromogen and then form a coloured compound in an acidic environment. The total number of oxidant molecules is associated with the colour intensity determined by spectrophotometry. The oxidative stress index (OSI) was obtained as a percentage of the ratio of TOS to TAS^(29)^.

### Statistical analysis

All data were evaluated using the SPSS 20·0 statistical package programme (Statistical Package for Social Sciences). Numbers and percentages were used for qualitative data. The compliance of all data with the normality distribution was evaluated analytically (Kolmogorov–Smirnov test) and visually (histogram graphs), and for normally distributed variables, parametric tests such as the Student’s *t* test and ANOVA were applied. Kruskal–Wallis and Mann–Whitney *U* tests were used for non-normally distributed variables. The daily nutrient and energy intakes were analysed by multiple logistic regression, adjusting for potential confounding factors. Statistical significance was set at *P* < 0·05.

## Results

A total of seventy individuals – thirty-five with RA and thirty-five with control – were included in this study. The distribution of individuals according to their general characteristics is given in Table 1. The average ages of the patients with RA and control were 45·4 ± 11·61 and 42·5 ± 8·50 years, respectively (*P* > 0·05). Patients with RA had lower total education time compared with healthy controls (*P* < 0·001). The BMI values of individuals in the RA and control groups were similar (*P* > 0·05). In the RA group, 25 % of the individuals had a normal BMI, 42·9 % were overweight and 31·4 % were obese. The average physical activity level of patients with RA (1·53 ± 0·13) was lower than the control group (1·59 ± 0·10) (*P* = 0·01). Individuals in both groups are classified as having a sedentary or light activity lifestyle according to the average physical activity level values. The average age of disease onset was 39·1 ± 11·47 years, and the average duration of RA was 6·4 ± 5·2 years, and the average DAS28 score was 3·2 ± 1·22 in the patient group. Of the patients, 48.·6 % had low disease activity, 45·7 % had moderate and only 5·7 % had high disease activity.

**Table 1.** General characteristics of individuals

Parameters	RA cases (*n* 35)		Healthy control (*n* 35)		*P*
	X	sd	X	sd	
Age (years)	45·4	11·61	42·5	8·50	0·23*
Total education time (years)	8·0	4·48	14·7	5·45	**<0·001** †
BMI (kg/m^2^)	27·4	4·9	26·7	4·57	0·49*
PAL	1·53	0·13	1·59	0·10	**0·01** *
DAS28	3·2	1·22	–		
Onset age of disease (years)	39·1	11·47	–		
Duration of disease (years)	6·4	5·20	–		
**BMI classification**	***n***	**%**	***n***	**%**	***P***
Normal	9	25·7	14	40·0	0·42‡
Overweight	15	42·9	11	31·4	
Obese	11	31·4	10	28·6	
**DAS28 classification**					
Low	17	48·6			
Moderate	16	45·7			
High	2	5·7			
**Treatment** §					
DMARD	35	100·0			
Biological agent	4	11·4			
Corticosteroids	22	62·8			
NSAID	5	14·3			

RA, rheumatoid arthritis; PAL, physical activity level; DMARD, disease-modifying anti-rheumatic drugs; NSAID, non-steroidal anti-inflammatory drug; DAS28, disease activity score-28. Data were presented as mean (X) and sd, or *n* (%), where appropriate.

* Independent-sample *t* test.

† Mann–Whitney *U* test.

‡ *χ* ^2^ test.

§ There is multiple drug use.

The evaluation of the disease activities of patients with RA according to BMI is shown in Table 2. The DAS28 score of patients with normal BMI was found to be lower (2·6 ± 1·13) than that of overweight (3·5 ± 1·17) and obese (3·3 ± 1·29) individuals, although this difference was not statistically significant. The number of swollen and tender joints in patients with RA was not different according to BMI classification (*P* > 0·05).

**Table 2.** Clinical findings of patients with RA according to BMI

Parameters	BMI classification						
	Normal (*n* 9)		Overweight (*n* 15)		Obese (*n* 11)		
	X	sd	X	sd	X	sd	*P*
DAS28 score	2·6	1·13	3·5	1·17	3·3	1·29	0·19*
Number of swollen joints	1·1	1·36	1·7	2·09	1·9	2·30	0·6†
Number of tender joints	1·0	2·0	1·0	2·00	1·0	3·00	0·60†
ESR (mm/h)	12·1	9·65	25·3	21·58	19·8	15·60	0·21†

RA, rheumatoid arthritis; DAS28, disease activity score28; ESR, erythrocyte sedimentation rate. Data were presented as mean and sd.

* ANOVA.

† Kruskal–Wallis.

It was observed that the serum TAS of the individuals in the control group (1·48 ± 0·16 mmol Trolox Equivalent) was higher than that of the RA group (1·40 ± 0·16 mmol Trolox Equivalent) (*P* = 0·05). The TOS and OSI values of patients with RA were found to be higher than the control group (*P* < 0·001) (Table 3).

**Table 3.** Serum total antioxidant, total oxidant status and oxidative stress index values of individuals

Parameters	RA cases (*n* 35)		Healthy control (*n* 35)		*P*
	X	sd	X	sd	
TAS (mmol Trolox Equivalent)	1·4	0·16	1·5	0·16	**0·05** *
TOS (µmol H_2_O_2_ Equivalent)	9·6	2·31	6·9	1·31	**<0·001** †
OSI	0·7	0·17	0·5	0·11	**<0·001** †

RA, rheumatoid arthritis; TAS, total antioxidant status; TOS, total oxidant status; OSI, oxidative stress index. Data were presented as mean and sd.

* Independent-sample *t* test.

† Mann–Whitney *U* test.

Table 4 illustrates the dietary energy and energy-adjusted nutrient intakes of participants. The daily intake of energy, MUFA, oleic acid and vitamin C were lower in the RA group compared with the control group, but the difference was not found to be statistically significant (*P* > 0·05). The daily protein and fibre intake (59·7 ± 18·52 g and 19·8 ± 6·3 g, respectively) of patients with RA was found to be lower compared with the control group (68·9 ± 17·17 g; 37·4 ± 12·62 g) (*P* = 0·03, *P* < 0·001, respectively). The daily intake of EPA, retinol, niacin, vitamin B_12_, Fe and Zn were lower in patients with RA than that of the healthy control. Individuals with higher fibre intake showed a significantly lower risk for RA after adjusting for potential confounding factors (OR = 0·845, 95 % CI = 0·773–0·923, *P* < 0·001). In addition, the average dietary total antioxidant intake was lower in the RA group (10·6 ± 2·60 mmol) than the control group (11·8 ± 1·99 mmol) (*P* = 0·04).

**Table 4.** Logistic regression analysis of daily dietary energy and energy-adjusted nutrient intake of individuals

Energy and nutrient intake	RA cases (*n* 35)		Healthy control (*n* 35)		*P*	Adjusted model		
	X	sd	X	sd		OR	95 % CI	*P*
Energy (kcal)	1641·2	447·58	1816·2	535·62	0·14*	1·000	0·999, 1·001	0·90
Protein (g)	59·7	18·52	68·9	17·17	**0·03** *	0·985	0·938, 1·035	0·56
Fibre (g)	19·8	6·30	37·4	12·62	**<0·001** †	0·845	0·773, 0·923	**<0·001**
MUFA (g)	23·7	8·90	29·3	13·23	0·09†	0·960	0·890, 1·037	0·30
PUFA (g)	20·8	9·52	21·6	9·53	0·73*	1·049	0·971, 1·133	0·23
Oleic acid (g)	21·9	8·47	27·2	12·61	0·08†	0·957	0·885, 1·035	0·27
ω-3 PUFA (g)	1·1	0·83	1·1	0·53	0·49†	1·811	0·608, 5·391	0·29
ω-6 PUFA (g)	19·5	9·25	20·2	9·08	0·75*	1·045	0·966, 1·130	0·27
EPA (g)	0·04	0·10	0·05	0·10	**0·006** †	0·653	0·003, 138·2	0·88
DHA (g)	0·1	0·16	0·1	0·17	0·31†	0·748	0·022, 25·100	0·87
Retinol (µg)	609·1	2126·02	1223·6	4016·71	**0·006** †	1·000	1·000, 1·000	0·53
Carotene (mg)	4·0	3·27	4·2	3·49	0·87†	1·036	0·868, 1·237	0·70
Vitamin C (mg)	128·4	99·11	128·5	85·17	0·77†	1·002	0·995, 1·008	0·62
Vitamin E (mg)	23·9	10·82	24·8	10·88	0·72*	1·009	0·948, 1·073	0·79
Niacin (mg)	11·6	5·52	16·0	9·87	**0·03** †	0·924	0·838, 1·019	0·11
Vitamin B_12_ (µg)	5·8	9·62	7·9	14·18	**0·01** †	1·000	0·954, 1·048	0·10
Ca (mg)	711·5	237·00	756·3	279·10	0·47*	1·003	1·000, 1·006	0·07
Fe (mg)	9·4	3·05	11·3	3·66	**0·02** †	0·840	0·677, 1·043	0·12
Zn (mg)	8·8	2·62	10·3	2·87	**0·02** †	0·748	0·534, 1·047	0·09
Dietary total antioxidant (mmol)	10·6	2·60	11·8	1·99	**0·04** *	0·799	0·623, 1·026	0·08

RA, rheumatoid. Data were presented as means and sd.

* Independent-sample *t* test.

† Mann–Whitney *U* test.

Logistic regression analysis for each nutrient included total education time and total energy intake in the adjusted model.

The results showed that the average Mediterranean diet adherence score of the RA group was lower than the control group (*P* = 0·003). Low adherence scores were found in 48·6 % of the RA group and 17·1 % of the control group (*P* = 0·01) (Table 5).

**Table 5.** Adherence to the Mediterranean diet score of individuals

MEDAS score	RA cases (*n* 35)		Healthy control (*n* 35)		*P*
	*n*	%	*n*	%	
≤ 5 (low)	17	48·6	6	17·1	**0·01** *
6–9 (medium)	18	51·4	27	77·2	
≥ 9 (high)	–	–	2	5·7	
**Mean and sd**	5·8	1·39	6·8	1·56	**0·003** †

RA, rheumatoid arthritis; MEDAS, Mediterranean diet adherence score.

* *χ* ^2^ test.

† Mann–Whitney *U* test.

## Discussion

Symmetric inflammation of freely moving joints is the hallmark of RA that can result in permanent pain, loss of function and disability. Due to the challenges in medical treatment and the side effects of drugs, supportive alternatives like nutritional therapy are increasingly needed^(1)^. The risk factors of RA are genetic and non-genetic such as smoking, altered microbiota and Western diet^(30)^.

The prevalence of RA in women peaks between the ages of 30 and 50 due to the influence of perimenopausal hormonal changes^(31)^. The average age of individuals with RA in this study was 45·4 ± 1161 years. The average education time of patients with RA was lower than that of the control group (*P* < 0·001). Similarly, Kroot *et al.* reported that the education level of patients with RA was lower than the country’s population in the Netherlands^(32)^.

Adipocytes play a significant role in regulating inflammation. Increased adiposity generally triggers pro-inflammatory molecules. Some meta-analyses have shown that obesity increases the risk of RA development^(33,34)^. It has been reported that in patients with RA, a high BMI is linked to a worse response to treatment. The moderate increase in serum TNF-α levels with body weight gain partially explains this relationship^(35)^. A recent study reported that 30·9 % of RA patients were overweight and 45·5 % were obese. While it has been reported that there is no correlation between the disease activities (DAS28) and BMI, a positive correlation was stated between detailed composition measurements such as % body fat and the number of swollen or tender joints^(36)^. Similarly, in our study, most of the individuals in the RA group were overweight (42·9 %) and obese (31·4 %). The DAS28 score of patients with normal BMI was found to be lower than that of obese patients but not statistically significant (*P* > 0·05). In addition, although not statistically significant, normal individuals’ DAS28 scores had low disease activity on average, while overweight and obese individuals had moderate disease activity. This shows that the clinical condition of patients with normal BMI is better than the others. Assessing and improving body composition in RA patients is crucial for the clinical course of the disease.

T cell and macrophage activation at the site of inflammation causes an increase in oxygen use, resulting in oxidative stress in RA. The joint damage develops as a result of elevated oxidative stress in RA^(4)^. In a recent study, serum α-tocopherol and enzymatic antioxidant levels of the control group were reported to be higher than those of the RA patient. Also, the decrease in serum antioxidant levels may be caused by both inadequate intake of antioxidant nutrients and excessive use of antioxidants due to inflammatory processes^(37)^. Several studies have reported that patients with RA exhibit significantly lower levels of enzymatic antioxidants, serum total antioxidants, vitamin C and glutathione, while showing higher levels of malondialdehyde level, the end product of lipid peroxidation, compared with healthy controls. Consequently, it has been reported that antioxidant levels in RA decrease to compensate for increased oxidative stress^(38,39)^. According to a recent study, total antioxidant capacity (ferric reducing antioxidant power) was higher in healthy controls compared with RA patients, while advanced glycation end products and advanced oxidation protein products were lower. They also stated that there was no significant relationship between advanced glycation end products, advanced oxidation protein, ferric reducing antioxidant power and disease activity^(40)^.

It was stated that evaluation of the total measurement instead of individual measurements to determine oxidative stress and antioxidant status in the body gives more meaningful results since the oxidants and antioxidants in the body interact with each other^(28,29)^. Demircan^(41)^ reported that the serum TAS was lower and TOS was higher in patients with RA than that of healthy controls, but not statistically significant. It was also stated that the OSI values of individuals with RA were significantly higher than the control group (*P* = 0·02). The DAS28 score and the TAS, TOS and OSI values were not correlated in RA patients. Prescha *et al.* ^(42)^ reported that the serum TAS was lower in RA patients than that of healthy controls (*P* = 0·03). Similar to the literature, we observed that the serum TAS value of individuals in the control group (1·4 ± 0·16 mmol Trolox Equivalent) was higher than that of the RA group (1·5 ± 0·16 mmol Trolox Equivalent) (*P* = 0·05). The serum TOS and OSI were lower in the control group than the RA group (*P* < 0·001). In this study, there was no correlation between serum TAS, TOS or OSI value and clinical parameters of patients with RA (*P* > 0·05) (data not shown). Kardeş *et al.* ^(43)^ reported that plasma non-enzymatic superoxide scavenging activity in RA was negatively correlated with the DAS28 score (*r* 0·396, *P* = 0·009), the number of swollen joints (*r* –0·342, *P* = 0·021) and the number of tender joints (*r* –0·304, *P* = 0·042), while malondialdehyde or superoxide dismutase were not related to these parameters. The heterogeneity of RA patients, the difficulty in achieving remission due to drug efficiencies and the use of different methods in assessing antioxidant capacity may have caused the differences between studies.

The aim of nutritional therapy for patients with RA is to increase antioxidant intake and decrease the ratio of ω-6 to ω-3 fatty acids in order to reduce inflammation. Anti-inflammatory diets have been shown to reduce pain in RA patients compared with regular diets^(30)^. Sufficient amounts of ω-3 PUFA intake reduce the formation of ω-6-induced pro-inflammatory eicosanoids. Therefore, it has been reported that especially EPA may protect against RA or decrease disease activity^(44)^. Giuseppe *et al.* ^(45)^ reported that long-term intake of 0·21 g/d of long-chain ω-3 PUFA reduced the risk of RA by 52 %, and consumption of ≥ 1 portion of fish per week was related to a 29 % decrease in the RA risk compared with consuming < 1 portion. However, there was no significant difference in PUFA and ω-3 intakes of groups; the EPA intakes of RA patients (0·04 ± 0·10 g) were found to be significantly lower than the control group (0·05 ± 0·10 g) (*P* = 0·006) in this study. In addition, a specific assessment of EPA intake would yield more significant results, as the average fish intake of the RA group was < 1 serving/week (not shown in the results).

A study reported that the protein, vitamin E, niacin, Fe and Zn intakes were significantly lower in RA patients than the control group^(37)^. Dietary energy, folic acid, vitamin B_6_, Ca, Mg and Zn intake were found to be lower than the requirement in RA patient in another study^(46)^. It was reported that the Zn intake (*P* < 0·001) and plasma Zn concentration (*P* = 0·04) were lower in RA patients than the control group. However, the disease activity of patients with RA did not affect biomarkers of Zn^(47)^. Similarly, we found that dietary protein, retinol, niacin, vitamin B_12_, Fe and Zn intake were lower in RA patients than that of the control group. A recent study indicated that encouraging a naturally high level of antioxidant capacity may help prevent the development of RA and showed that there was a negative correlation between dietary total antioxidant capacity and the risk of RA^(48)^. In this study, the dietary total antioxidant intake was found to be lower in the RA group than the control group (*P* = 0·04). In patients with RA, adequate intake of antioxidant and anti-inflammatory nutrients is very important for maintaining oxidative balance. Therefore, it is important to evaluate the nutritional status of these individuals and replace the inadequate nutrients.

It has been reported that the lower incidence of RA in southern European nations compared with northern European nations cannot be attributed solely to racial and genetic differences. Environmental factors and lifestyle, including diet, also play a significant role in this difference. It has been stated that the Mediterranean diet may result in lower levels of disease activity, inflammatory markers and cardiovascular risk factors by regulating the lipid profile, increasing antioxidant levels, reducing inflammation and changing intestinal flora^(49)^. In addition, consumption of vegetables, fruits and legumes, which are components of the Mediterranean diet, is negatively associated with the disease score^(15)^. A recent systematic review demonstrated that the administration of omega-3 PUFA, fish oil and adherence to the Mediterranean diet can decrease measures of disease activity^(50)^. The average Mediterranean diet adherence score of the RA group (5·8 ± 1·39) was found to be lower than the control group (6·8 ± 1·56) (*P* = 0·003) in this study. Matsumoto *et al.* ^(51)^ did not show any differences between the Mediterranean diet adherence scores of RA patients and healthy individuals. The study reported that the median Mediterranean diet score of individuals was 4 point. The low adherence seen in this study may have affected the study results due to the change in eating behaviour of the countries. However, it has been reported that vegetable consumption and MUFA intake, which are two of the basic components of the Mediterranean diet, are lower in patients with RA, and it has been stated that the main factor in the effectiveness of the Mediterranean diet may be related to MUFA intake^(51)^. Dietary fibre intake provides the anti-inflammatory properties of the Mediterranean diet by the influence on the metabolic activity and composition of the gut microbiota. It has been reported that higher dietary fibre intake increases the anti-inflammatory SCFA and alters the gut microbiome composition^(52)^. We observed that dietary fibre intake in RA patients was lower compared with the control group (*P* < 0·001). In addition, after adjusting for potential confounding factors such as energy intake and total education time, higher fibre intake was associated with a lower risk for RA (OR = 0·845, 95 % CI = 0·773–0·923, *P* < 0·001).

A randomised clinical trial reported that the combination of a Mediterranean diet and a dynamic exercise programme could increase the quality of life in RA patients with low disease activity^(53)^. RA is associated with significant functional losses and increased CVD risk. Physical activity and exercise are very important in reducing joint problems such as pain and aches and the increased risk of CVD by reducing C-reactive protein and IL-6 levels and suppressing inflammation in RA^(54)^. Several studies have shown that the physical activity levels of healthy individuals were higher than those of patients with RA^(48,55)^. Similarly, the mean physical activity level of individuals in the RA group was found to be lower than the control group in this study (*P* = 0·01). In a randomised clinical trial, it was reported that a Mediterranean diet and dynamic exercise programme increased hand grip strength and the health assessment questionnaire disability index and decreased waist circumference^(56)^.

In conclusion, this study has demonstrated that patients with RA had lower serum TAS and higher TOS compared with healthy individuals. We have also observed that the Mediterranean diet can be an adjuvant to medical treatment and positively influence the disease process by reducing oxidative stress. In addition, patients’ participation in physical activity adjusted to their physical functions should be increased. To learn more about how nutrition affects inflammation and whether the Mediterranean diet may benefit RA patients, more clinical research is required.
